# Ambulatory oral surgery: 1-year experience with 11 680 patients from Zagreb district, Croatia

**DOI:** 10.3325/cmj.2013.54.49

**Published:** 2013-02

**Authors:** Dražen Jokić, Darko Macan, Berislav Perić, Marinka Tadić, Josip Biočić, Petar Đanić, Davor Brajdić

**Affiliations:** 1 Department of Oral and Maxillofacial Surgery, University Hospital Dubrava, School of Dental Medicine, University of Zagreb, Zagreb, Croatia; 2 Public Health Clinic “Centar,” Zagreb, Zagreb, Croatia

## Abstract

**Aim:**

To examine the types and frequencies of oral surgery diagnoses and ambulatory oral surgical treatments during one year period at the Department of Oral Surgery, University Hospital Dubrava in Zagreb, Croatia.

**Methods:**

Sociodemographic and clinical data on 11 680 ambulatory patients, treated between January 1 and of December 31, 2011 were retrieved from the hospital database using a specific protocol. The obtained data were subsequently analyzed in order to assess the frequency of diagnoses and differences in sex and age.

**Results:**

The most common ambulatory procedure was tooth extraction (37.67%) and the most common procedure in ambulatory operating room was alveolectomy (57.25%). The test of proportions showed that significantly more extractions (*P* < 0.001) and intraoral incisions (*P* < 0.001) were performed among male patients, whereas significantly more alveolectomies and apicoectomies were performed among female patients (*P* < 0.001). A greater prevalence of periodontal disease was found in patients residing in Zagreb than in patients residing in rural areas.

**Conclusion:**

The data from this study may be useful for planning of ambulatory oral surgery services, budgeting, and sustaining quality improvement, enhancing oral surgical curricula, training and education of primary health care doctors and oral surgery specialists, and promoting patients’ awareness of the importance of oral health.

Oral health is essential to general health and well-being, and tooth loss is an important indicator of oral health status. Reduction of tooth loss is one of the targets for achieving global goals for oral health (1).

Croatian dental health system, along with general health care, is regulated by the Health Care Law and divided into three levels of care: primary, secondary, and tertiary. Primary dental health care is performed by doctors of dental medicine with the help of dental assistants; it includes prevention, detection, and treatment of oral diseases, as well as rehabilitation of the masticatory system. Secondary or specialist outpatient dental health care treats patients in need of expert opinion, advice, or treatment. Tertiary care provides care only for patients requiring hospitalization.

In order for the system to work properly and without overload, levels of health care must cooperate. Most patients with oral needs visit a primary health care system dentist, so further education of these professionals is essential for a better system of specialist referrals and a more efficient health care system. In Croatia, there are no data about the types and frequencies of oral surgery treatment so the aim of this study was to gain insight into the types and frequencies of diagnoses and ambulatory surgical treatments at the Department of Oral Surgery at University Hospital Dubrava in Zagreb, Croatia. We also investigated whether there was a significant difference in clinical diagnoses and procedures according to sex, age, and place of residence.

## Materials and methods

A total of 11 680 patients admitted to the Department of Oral Surgery, University Hospital Dubrava from January 1 to December 31, 2011 were included in the study. Among these, 3447 where scheduled for surgical procedures under local anesthesia in the ambulatory outpatient operating room and 8233 received surgical procedures in the ambulatory outpatient clinic, where new patients are seen, diagnosis is made, and a treatment plan is formulated. A total of 2201 patients indeed received surgical procedures in the ambulatory outpatient operating room and these were the patients included in the study.

The data were collected retrospectively from both ambulatory outpatient clinic protocols and ambulatory outpatient operation protocols. To digitize the data more easily we used two forms designed by Microsoft Access 2007 and they comprised all the data that could be gathered from the Department protocols. The form for the ambulatory clinic protocols classified the patients according to the date of arrival, age, sex, place of residence, and clinical diagnosis. After data collection, diagnoses and surgical treatments were categorized to facilitate statistical analysis.

We also investigated whether there was a difference in clinical diagnoses and procedures between patients residing in Zagreb and those residing in rural areas. We divided the patients into three groups according to the place of residence (group 1: Zagreb; Group 2: patients from settlements with fewer than 10 000 residents; group 3: patients from cities with more than 10 000 residents excluding Zagreb). Data were obtained from the Croatian Census 2011 (2).

Statistical calculations were made using SPSS 19 (SPSS Inc, Chicago, IL; USA). We used continuous (age) and categorical data (sex, place of residence, clinical diagnosis, and treatment). Age was presented as means and standard deviations and age differences were compared using independent samples *t* test. Categorical variables were presented as absolute and relative frequencies, and were compared using a χ^2^ test of proportions. *P* value lower than 0.05 was considered to be statistically significant.

## Results

The study included 5272 (45.14%) men and 6408 (54.86%) women, with mean age 38.7 ± 19.4 years. Of the 2201 patients who underwent ambulatory operations under local anesthesia, 870 were men (39.52%) and 1331 were women (60.48%), with mean age 35.5 ± 16.2 years. Alveolectomies and frenulectomies were performed mostly on younger patients (33.6 ± 14.5 years), while patients who were treated with remodeling of the alveolar ridge, excisions, and implantations were mostly elderly (51.8 ± 17.5 years). The mean age of the patients scheduled for surgical procedures (32.9 ± 16.9 years) was lower than that of those who underwent surgery (35.5 ± 16.2 years). The reason for this is that patients under the age of 7 were scheduled for hospitalization and surgery under general anesthesia, which was not included in this survey (Table 1). It is also interesting that there were 1246 patients who were scheduled for surgery but they did not show up.

**Table 1:** The number and age of patients and who underwent procedures in the outpatient ambulatory clinic

Procedure	No. of patients (%)	Age in years (mean ± standard deviation)
Tooth extraction	4400 (37.67)	41.7 ± 19.9
Examination and scheduled for surgery	3447 (29.51)	32.9 ± 16.9
Examination	2211 (18.93)	42.0 ± 20.3
Follow-up examination	1259 (10.78)	39.0 ± 19.3
Intraoral incision	159 (1.36)	36.3 ± 17.7
Other	204 (1.74)	34.8 ± 16.2

We analyzed the frequency of clinical diagnoses (Table 2). There were 77 different clinical diagnoses in the protocol but only ten diagnoses appeared in more than 1% of all patients. Retained roots, chronic periapical lesions, and deep caries represented 70.37% of all ambulatory diagnoses. We also determined the number of clinical procedures performed in the ambulatory operating room (Table 3). The category “other diagnoses” included 34 diagnoses that occurred in fewer than five patients.

**Table 2:** Clinical diagnoses in the outpatient ambulatory clinic

Clinical diagnosis	No. of patients	(%)
Retained root	2378	45.55
Chronic periapical lesion	658	12.60
Deep caries	638	12.22
Periapical abscess	336	6.44
Periodontal disease	205	3.93
Teeth trauma	194	3.72
Temporomandibular joint disorders	145	2.78
Semi-impacted third molars	139	2.66
Pericoronitis	78	1.49
Oral and maxillofacial trauma	59	1.13
Complications after tooth extractions	46	0.88
Cysts	45	0.86
Impacted teeth	48	0.92
Retained (embedded) teeth	36	0.69
Trigeminal neuralgia	31	0.59
Atrophy of alveolar ridge	27	0.52
Anodontia	24	0.46
Exostosis	23	0.44
Mucosal hypertrophies	23	0.44
Oroantral communication	12	0.23
Epulis	12	0.23
Mucocele	10	0.19
Periapical fistula	10	0.19
Frenulum breve	9	0.17
Disease of pulp	9	0.17
Lymphadenitis	7	0.13
Sialoadenitis	7	0.13
Oroantral fistula	6	0.11
Foreign body	3	0.06
Mucosal hemangioma	3	0.06
Total	**5221**	**100.00**

**Table 3:** Clinical diagnoses in the outpatient ambulatory operating room

Clinical diagnosis	No. of patients	(%)
Impacted teeth	740	33.62
Chronic periapical lesion	435	19.76
Retained root	362	16.45
Cysts	159	7.22
Retained (embedded) teeth	72	3.27
Deep caries	67	3.04
Persistent tectolabial frenulum	59	2.68
Fibroma	39	1.77
Irregular alveolar process	38	1.73
Mucosal hypertrophies	26	1.18
Pericoronitis	25	1.14
Teeth trauma	24	1.09
Epulis	23	1.04
Mucocele	20	0.91
Anodontia	12	0.55
Oroantral fistula	12	0.55
Tumor	9	0.41
Ankyloglossia	8	0.36
Displaced root in sinus	7	0.32
Oroantral communication	6	0.27
Foreign body	5	0.23
Other diagnosis	53	2.41
Total	**2201**	**100.00**

The study included surgical treatments in the ambulatory clinic and ambulatory operating room. Tooth extraction was the most common ambulatory procedure. Alveolectomy was the most common procedure performed in the ambulatory operating room and, together with apicoectomy, amounted to 73.97% of the total number of operations (Table 4). The category entitled “other treatment” included treatments done fewer than 50 times. There was significantly more tooth extractions (39.98% vs 35.77%, *P* < 0.001) and intraoral incisions (1.84% vs 0.97%, *P* < 0.001) among male patients, whereas women were more often scheduled for surgery (Table 5).

**Table 4:** Surgical treatments under local anesthesia in the outpatient ambulatory operating room (treatments performed more than 50 times)

Surgical treatment	Number	(%)
Alveolectomy	1260	57.25
Apicoectomy	368	16.72
Cystectomy	147	6.68
Excision	113	5.13
Frenulectomy	67	3.04
Exploration	59	2.68
Other	187	8.50
Total	2201	100.00

**Table 5:** Ambulatory procedures in outpatient clinic and outpatient ambulatory operating room according to sex

Procedures	No. (%) of patients	χ^2^	*P*


male	female
Tooth extraction	2108 (39.98)	2292 (35.77)	21.91	<0.001
Examination and scheduled for surgery	1417 (26.88)	2030 (31.68)	32.05	<0.001
Examination	995 (18.87)	1216 (18.98)	0.02	0.890
Follow-up examination	573 (10.87)	686 (10.71)	0.08	0.770
Intraoral incision	97 (1.84)	62 (0.97)	16.40	<0.001
Other	82 (1.56)	122 (1.90)	2.04	0.150
Total	5272 (100.00)	6408 (100.00)		

Of 11 680 patients, 6597 (56.48%) were from Zagreb, 3292 (28.18%) were from rural areas, and 1791 (15.34%) were from other cities. Patients from Zagreb had significantly greater incidence of periodontal diseases (χ^2^ = 4.44; *P* = 0.035). They also underwent tooth extractions significantly more often than patients from rural areas (χ^2^ = 37.69; *P* < 0.001), who were more frequently scheduled for surgery (χ^2^ = 10.65; *P* = 0.001). Rural regions and Zagreb did not show significant differences in other diagnoses or clinical treatment.

## Discussion

In our study, 11 680 patients were treated in oral ambulatory outpatient clinic and 2201 were operated on under local anesthesia in ambulatory outpatient operating room. The first study assessing the number of procedures performed in the ambulatory Department of Oral Surgery was published in 1958 (3). In the period between 1971 and 1982, the average number of annual ambulatory oral surgical procedures under local anesthesia was 584. Since that period, the number of surgeries has increased more than three and a half times (4). In 1990, 2025 surgeries under local anesthesia were carried out. In the same year, 12 374 patients were treated as outpatients at the ambulatory clinic. The number of surgical procedures in 2011 found in our study was the greatest in the history of the department. Because oral surgery and maxillofacial surgery in Croatia are separate specialties, we selected the results from the available literature from maxillofacial departments, but from the domain of oral surgeons (5,6).

Ćabov et al found that surgically treated patients (7) had an age range of 5-88 years, with a median age of 37 years for men and 31 years for women. These numbers do not substantially deviate from other literature data (8,9). Our patients also had a similar age range. However, patients who were scheduled for surgery had an age-range from 1 to 85 years. The reason for this is that the youngest age groups were scheduled for surgery under general anesthesia and were not included in the study.

In our study, more women underwent surgery, which is in accordance with previous findings (4). This could be a result of an increased prevalence of caries among women. Women had more alveolectomies and apicoectomies, while men had more tooth extractions and intraoral incisions. A study in North India (10) also found that women had more impacted teeth. Similarly, an analysis of emergency cases in oral surgery showed that the main reasons for admission were acute odontogenic infections, which are more often seen in men (11). This could suggest that women are more aware of oral health than men.

The proportions of certain surgical procedures have changed over the years. In the 1960s, alveolectomies represented 45% and apicoectomies 19% of all treatments (3). By 1991, the share of apicoectomies grew to 40.1% (4) and from 1995 to 2000 they comprised between 30 and 40% of all services (11). We found the lowest share of apicoectomies so far (only 16.72%). However, due to the increase in the overall number of surgical procedures, the number of apicoectomies is actually higher than ever; teeth that were once indicated for extraction are now apicoectomised. Dhariwal et al (12) reported a decrease in the number of apicoectomies in England and Wales for the period between 1991 and 2000. They found a correlation with the overall incidence of caries and endodontic treatments, which also dropped.

The number of alveolectomies performed in the department from the 1960s to 2007 ranged from 45% to 57.2% (3,7,11). Arole (5) reported only 560 (18.8%) alveolectomies performed as day-case surgery between 1987 and 1997. Barry et al (6) reported an increase in alveolectomies in one-day surgeries from 10.5% to 57% between 1968 and 1992 and Thomas et al (13) found an increase in the number of alveolectomies in the period between 1984 and 1991.

Gilhorpe et al (14) found that the most common oral surgery procedure in England and Wales were apicoectomies, with the frequency of 6.2%, which is much lower than in our study. Our results are similar to the results of Lyons et al (15) from the District General Poole Hospital, England, who found the frequency of 15%. In Ontario Canada, the most frequently performed day surgery procedure were extractions (16). They also found that approximately half of the visits to emergency department for dental care were associated with periapical abscesses and toothaches (17).

In the Dental Clinic of the University of Barcelona, 15.34% of oral surgery treatments were performed on patients younger than 18 years of age (18). In our study, this number was significantly lower (10.13%). The reason for this is that younger patients were scheduled for surgery under general anesthesia, which is not included in this survey

Our study found a significantly higher proportion of patients with periodontitis among Zagreb residents than among rural areas residents. Similarly, Ćabov et al (7) found a higher prevalence of periodontal disease in an urban environment. However, our results should be interpreted with caution because the criteria for periodontal disease in our study might not have been applied carefully in all patients who came to ambulatory department of oral surgery. Future studies should examine the education level of patients, which could have important impact on the incidence of periodontal disease. Ćabov et al also found an increased incidence of cysts in patients from rural areas, leading to the need for preprosthetic surgery. Kalyanpur and Prasad (19) found that rural residence, increased age, and female sex were related to teeth mortality rates caused by dental caries and periodontal disease.

Chrysanthakopoulos (20,21) reported 2418 extracted permanent teeth during a period of 5 years. Baqain et al (22) found 2435 patients seeking help in Oral Surgery Clinic over a 3-year period. It seems that they had almost 5 times fewer patients and almost 10 times fewer extractions per year than we did.

In Croatia, Peršić et al (23) found that patients 31-40 years old had 12.2% diseased endodontically treated teeth, while Matijević et al (24) found that 75.9% of participants had endodontically treated teeth and 8.5% of all teeth were endodontically treated. Periapical lesions were detected in 8.5% of teeth, and 65.8% of endodontically treated roots had inadequate root canal filling length. Dukić et al (25) found a high caries prevalence among schoolchildren in Zagreb, although dental caries is decreasing in many European countries. Because of poor oral health and low oral hygiene as well as unsatisfactory prevention in Croatia, a considerable number of these patients will seek help in oral surgery outpatient clinics. Deep caries, chronic periapical lesions, and periapical abscess were found in more than one third of our patients, which largely explains the increasing number of patients in our outpatient department.

The most common clinical diagnoses in our outpatients were retained roots, chronic periapical lesions, and deep caries. Also, we found a significant increase in the share of alveolectomies and a reduction in the share of apicoectomies performed in the ambulatory operating room compared to other studies. Tooth extractions, which were the most common surgical treatment, and intraoral incisions are the procedures that could be done by every primary health care dentist; as such, improved training of dentists and dental students could result in fewer referrals to oral surgeons. Also, the number of surgical procedures could be reduced by better education of patients on the importance of oral health and regular checkups. Therefore, the results of this study could be useful for planning of ambulatory oral surgery services, budgeting, and sustaining quality improvement, enhancing oral surgical curricula, training and education of primary health care doctors and oral surgery specialists, and promoting patients’ awareness of the importance of oral health.

## References

[1] Perera R, Ekanayake L (2011). Tooth loss in Sri Lankan adults.. Int Dent J.

[2] Croatian Bureau of Statistics. Census of population, households and dwellings, 2011. Available from: http://www.dzs.hr/Hrv/censuses/census2011/xls/Tab2_HR.xls Accessed: January 31, 2013.

[3] Amšel V, Knežević G (1968). Statistical review of surgical interventions in out-patients covering the period 1958-1968. Acta Stomatol Croat.

[4] Macan D, Kobler P, Knežević G, Grgurević J, Švajhler T, Krmpotić I (1991). Comparison of clinical and histopathological diagnosis in oral surgery. Acta Stomatol Croat.

[5] Arole G (1998). Day-case oral and maxollofacial surgery in a Nigerian district general hospital: scope and limitations.. Ann R Coll Surg Engl.

[6] Barry HJ, Sleeman D, Ryan CD, Allen FJ (1996). Oral surgery at St. Mary's Hospital, Chapelizod. A quarter century experience of day-case oral surgery.. J Ir Dent Assoc.

[7] Ćabov T, Filipović-Zore I, Kobler P, Dorčić D (2002). Epidemiological analysis of oral surgery procedures.. Coll Antropol.

[8] Adebayo ET, Ajike SO, Abite MG (2008). Audit of oral and maxillofacial surgical conditions seen at Port Harcourt, Nigeria.. Ann Afr Med.

[9] Varenne B, Msellati P, Zoungrana C, Fournet F, Salem G (2005). Reasons for attending dental-care services in Ouagadougou, Burkina Faso.. Bull World Health Organ.

[10] Jena AK, Duggal R, Parkash H (2010). The distribution of individual tooth impaction in general dental patients of Northern India.. Community Dent Health.

[11] Brajković B, Macan D (2002). Analysis of emergency cases in the Clinic of maxillofacial and oral surgery, Clinical Hospital Dubrava.. Acta Stomatol Croat.

[12] Dhariwal DK, Goodey R, Shepherd JR (2002). Trends in oral surgery in England and Wales 1991-2000.. Br Dent J.

[13] Thomas D, Walker R, Smith A, Shepherd J (1994). The provision of oral surgery services in England and Wales 1984-1991.. Br Dent J.

[14] Gilthorpe MS, Wilson RC, Bedi R (1997). A sociodemographic analysis of inpatient oral surgery: 1989-1994.. Br J Oral Maxillofac Surg.

[15] Lyons AJ, Hughes CE, Dixon EJ (1995). A 5-year audit of outcome of apicectomies carried out in a district general hospital.. Ann R Coll Surg Engl.

[16] Quinonez C, Gibson D, Jokovic A, Locker D (2009). Day surgery visits for dental problems.. Community Dent Oral Epidemiol.

[17] Quinonez C, Gibson D, Jokovic A, Locker D (2009). Emergency department visits for dental care of nontraumatic origin.. Community Dent Oral Epidemiol.

[18] Perez-Garcia S, Chaparro-Avendano AV, Delgado-Molina E, Berini-Aytes L, Gay-Escoda C (2005). Day case oral surgery in pediatric patients during the year 2000 in the University of Barcelona Dental Clinic (Spain).. Med Oral Patol Oral Cir Bucal.

[19] Kalyanpur R, Prasad KV (2011). Tooth mortality and prosthetic needs among the urban and rural adult population of Dharwad district, India.. Oral Health Prev Dent..

[20] Chrysanthakopoulos NA (2010). Periodontal reasons for tooth extraction in adult population in Greece.. Acta Stomatol Croat.

[21] Chrysanthakopoulos NA (2011). A survey of the reasons for dental extraction in adult population in Greece.. Acta Stomatol Croat.

[22] Baqain ZH, Khraisat A, Sawair F, Ghanam S, Shaini FJ, Rajab L (2007). Dental extraction for patients presenting at Oral Surgery Student Clinic.. Compend Contin Educ Dent.

[23] Persic R, Lumnije K, Brumini G, Husetic M, Pezelj-Ribaric S, Brekalo-Prso I (2011). Difference in the periapical status of endodontically treated teeth between the samples of Croatian and Austrian adult patients.. Croat Med J.

[24] Matijevic J, Cizmekovic-Dadic T, Prpic-Mehicic G, Anic I, Slaj M, Jukic-Krmek S (2011). Prevalence of apical periodontitis and quality of root canal fillings in population of Zagreb, Croatia: a cross-sectional study.. Croat Med J.

[25] Dukic W, Delija B, Lulic-Dukic O (2011). Caries prevalence among schoolchildren in Zagreb, Croatia.. Croat Med J.

